# CD10 is a marker for cycling cells with propensity to apoptosis in childhood ALL

**DOI:** 10.1038/sj.bjc.6600329

**Published:** 2002-06-05

**Authors:** G Cutrona, P Tasso, M Dono, S Roncella, M Ulivi, E M Carpaneto, V Fontana, M Comis, F Morabito, M Spinelli, E Frascella, L C Boffa, G Basso, V Pistoia, M Ferrarini

**Affiliations:** Servizi di Immunologia Clinica, Istituto Nazionale per la Ricerca sul Cancro, IST, Genoa, Italy; Oncologia Sperimentale, Istituto Nazionale per la Ricerca sul Cancro, IST, Genoa, Italy; Epidemiologia Ambientale e Biostatistica, Istituto Nazionale per la Ricerca sul Cancro, IST, Genoa, Italy; Laboratorio di Oncologia, Istituto G Gaslini, Genoa, Italy; Dipartimento di Oncologia, Biologia e Genetica, Università di Genova, Genoa, Italy; Servizio di Istologia e Anatomia Patologica, Ospedale Sant' Andrea, La Spezia, Italy; TIB-Mol Biol, Berlin, Germany; Divisione di Ematologia, Dipartmento di Ematologia, Azienda Ospedaliera Bianchi-Melacrino-Morelli, Reggio Calabria, Italy; Centro Trapianti Midollo Osseo, Dipartimento di Ematologia, Azienda Ospedaliera Bianchi-Melacrino-Morelli, Reggio Calabria, Italy; Laboratorio di Emato-Oncologia, Azienda Ospedaliera, Università Padova, Padua, Italy; Dipartimento di Scienze Pediatriche Torino, Turin, Italy

**Keywords:** B lymphocytes, apoptosis, cell surface molecules, cellular proliferation

## Abstract

CD10 constitutes a favourable prognostic marker for childhood acute lymphoblastic leukaemia. Since correlations between CD10, cell cycle and apoptotic abilities were demonstrated in various cell types, we investigated whether differences existed in the cycling/apoptotic abilities of CD10-positive and CD10-negative B acute lymphoblastic leukaemia cells. Twenty-eight cases of childhood acute lymphoblastic leukaemia (mean age of 6.8 years) were subdivided into two groups according to high (17 cases, 93.2±4.5%, MRFI 211±82 CD10-positive cells) or low (11 cases, 11.5±6.2%, MRFI 10±7 CD10-negative cells) expression of CD10. CD10-positive acute lymphoblastic leukaemia cells were cycling cells with elevated c-*myc* levels and propensity to apoptosis, whereas CD10-negative acute lymphoblastic leukaemia cells had lower cycling capacities and c-*myc* levels, and were resistant to apoptosis *in vitro*. A close correlation between all these properties was demonstrated by the observations that the few CD10-positive cells found in the CD10-negative acute lymphoblastic leukaemia group displayed elevated c-*myc* and cycling capacities and were apoptosis prone. Moreover, exposure of CD10-positive acute lymphoblastic leukaemia B cells to a peptide nucleic acid anti-gene specific for the second exon of c-*myc* caused inhibition of c-*myc* expression and reduced cell cycling and apoptotic abilities as well as decreased CD10 expression.

*British Journal of Cancer* (2002) **86**, 1776–1785. doi:10.1038/sj.bjc.6600329 www.bjcancer.com

© 2002 Cancer Research UK

## 

Malignant B cells from the majority of childhood acute lymphoblastic leukaemia (ALL) cases are characterised by the surface expression of CD10, previously known as common acute lymphoblastic leukaemia antigen or CALLA (as reviewed in LeBien and McCormack, 1989; Shipp and Look, 1993; Weir and Borowitz, 2001). Although initially considered a tumour specific antigen, CD10 was subsequently detected on a variety of normal cells of haemopoietic and non haemopoietic origin. CD10, also called neutral peptidase 24.11 or NEP, possesses a well-defined enzymatic activity, but its function in the physiology of lymphoid cells is largely unknown. From the standpoint of childhood ALL, CD10 expression represents a favourable, although not independent, prognostic marker (Pui *et al*, 1993; Consolini *et al*, 1998). The reason as to why a surface molecule has impact on prognosis is not known, although a number of observations on other cell types suggest that CD10 may mark cells with special cycling and apoptotic abilities. For example, CD10 was found on the surface of cells that are particularly prone to apoptosis such as normal germinal centre B cells (Liu *et al*, 1992) or malignant cells from Burkitt's Lymphomas (BL) (Rowe *et al*, 1987). Moreover, it was observed that CD10 was expressed by T cells induced into apoptosis by a variety of means *in vitro* (Cutrona *et al*, 1999). Finally, c-*myc* upregulation not only induces cell entry into the early phases of cell cycle, but also renders the cells apoptosis-prone and concomitantly induces CD10 expression (Cutrona *et al*, 1995, 2000).

Based on the above observations, we reasoned that expression of CD10 by malignant B cells from ALL might indicate a special cycling ability as well as a propensity to undergo apoptosis. Indeed, in this study we demonstrate that expression of CD10 marks malignant ALL cells that are apoptotis-prone and actively cycling and express high levels of the c-*myc* oncogene. By contrast, CD10-negative ALL cells have lower c-*myc* levels and inferior cycling and apoptotic properties under the same *in vitro* conditions. CD10-positive ALL comprise different subgroups characterised by distinctive cytogenetic abnormalities. Nevertheless all of these cells appear to share the same cycling/apoptotic features, which can represent a common prognostic factor.

## MATERIALS AND METHODS

### Patients

Bone marrow aspirates of 28 cases of childhoood pre-B ALL (mean age of 6.8 years) were obtained from hospitals affiliated with the Italian Association of Pediatric Hematology and Oncology (AIEOP). Diagnosis of B ALL was based on morphological analysis of bone marrow aspirates according to the French–American–British (FAB) guidelines (Bennett *et al*, 1981), and on cytochemical and phenotypic features of the leukaemia cells. Such features include positive nuclear staining for terminal deoxynucleotidil transferase, negative staining for myeloperoxidase, expression of B-cell differentiation markers such as CD19 and cytoplasmic Ig μ chains and absence of surface Ig. Karyotype analysis did not reveal any chromosomal abnormality in which the c-*myc* oncogene could be involved, i.e. t(8; 14), t(2; 8), or t(8; 22) (Magrath, 1990). At the time of study, ALL patients were at the onset of disease and were untreated. All the bone marrow samples were stored in liquid nitrogen (−180°C) until tested. In the experiments with PNAs (see below), freshly prepared cells from cases #655, 657, 659, 660 and 661 were employed.

### Cytogenetic analysis

Bone marrow aspirates were processed as previously described (Sainati *et al*, 1997). The cytogenetic studies were performed using Trypsin–Giemsa banding technique. Chromosomes were identified and assigned according to the International System for Human Cytogenetic Nomenclature (Mitelman, 1995).

### Detection of specific chromosome gene aberration by RT–PCR

Total RNA was isolated by using the RNAzol-B reagent according to the manufactory protocol (Duotech srl Milan, Italy). Two micrograms of total RNA from each specimen was reversed transcribed by using the Superscript reverse trascriptase (Life Technologies Milan, Italy) and random examers: PCR amplification was performed using Amplitaq polymerase (Applied Byosistem) according to BIOMED-1 (van-Dongen *et al*, 1999) protocols. A screening for the following fusion gene transcripts t(1;19) with E2A-PBX1, t(4;11) with MLL-AF4, t(9;22) with BCR-ABL p190 and BCR-ABL p210, t(12;21) with TEL-AML1, was performed. An indipendent PCR reaction was performed with shift primers for confirmation of each positive result. The ABL housekeeping gene expression was assessed to determine the presence of amplificable RNA and the efficacy of reverse transcriptions. The electrophoresed PCR reaction products were stained with ethidiun bromide.

### Cells and cell cultures

Mononuclear cell fractions were purified from bone marrow aspirates by centrifugation on Ficoll-Hipaque gradients (Seromed, Biochrom KG, Berlin, Germany). All of the suspensions were comprised of more than 90% leukaemia cells except for cases #738 and 702, in which there were 55 and 69% leukaemic cells, respectively. Where indicated, special staining procedures had to be employed for these cases.

The LAM C4 cell line, derived from a Burkitt lymphoma patient, with the typical t(8; 14) translocation and c-*myc* over-expression was used as a positive control in the studies on c-*myc* expression (Roncella *et al*, 1993).

The culture medium used throughout was RPMI 1640 (Seromed) supplemented with 10% FCS (Seromed).

### Flow-cytometry

The following mAbs were used for immunofluorescence staining: anti CD10 (J5) (Coulter Corp., Hielah, FL, USA), and anti CD19 (Leu-12) (Becton Dickinson & Co., Sunnyvale, CA, USA). Both of these mAbs were used in indirect immunofluorescence. The secondary FITC- or PE-conjugated antibodies to the appropriate murine Ig isotype were from Southern Biotechnology (Birmingham, AL, USA). Permeabilised cells were stained for indirect immunofluorescence with a murine anti Myc mAb (6E10 clone, Cambridge Research Biochemicals, Cheshire, UK), an anti Ki67 mAb (DAKOPATTS, Glostrup, Denmark), and an anti Ig μ chains (Becton Dickinson & Co.) as previously reported (Cutrona *et al*, 1997). The samples were analysed by flow cytometry (FACS Calibur, Becton Dickinson & Co.). Data were expressed as histograms of fluorescence intensity *vs* cell number or as relative fluorescence intensity (MRFI) calculated according to the following formula: mean fluorescence intensity of cells stained with the mAb/mean fluorescence intensity of control cells treated with an unrelated mAb.

Two-colour analysis of 5-bromo-deoxyuridine (BrdUrd) (Sigma Aldrich, Milan, Italy) incorporation and DNA content was performed according to a modification of the method of Dolbeare *et al* (1985) as previously described (Dolbeare *et al*, 1985; Cutrona *et al*, 1997).

CD10 positive and negative cells from CD10-negative ALL cases were separated by cell sorting (FACS Calibur, Becton Dickinson & Co.). The two populations were gated on the basis of CD10 expression and the forward light scatter parameter.

Measurements of ploidy (DNA index, DI) was carried out by staining samples with a hypotonic solution of PI using an automated DNA staining device (DNA-prep reagents; Coulter) and analysed by flow cytometry (Epics XL; Coulter). DNA histograms were obtained by a cell cycle analysis program (Multicycle; Phoenix, San Diego, CA, USA). Leukaemic cells were classified as diploid when the DI was between 0.9 and 1.09; as hyperdiploid when the DI was 1.8 and less then 1.80; and as hypodiploid when DI was <0.9. The DI was established from the ratio of the modal channel number of the G0/G1 peak of neoplastic cells to that of normal cells.

### Western blot

Western blot analysis for MYC protein was performed as previously described (Cutrona *et al*, 1995). The nitrocellulose membranes were immunoblotted with an anti Myc hybridoma supernatant (9E10 clone, kindly provided by Dr R Sitia, San Raffaele Institute, Milan, Italy) or a rabbit antiserum to histone 2b (H2b) (kindly provided by Dr U Pfeffer, CBA, Genoa, Italy).

### PCR methodologies

The PCR technique for analysis of V(D)J rearrangements and of HCDR3 length was performed as previously described (Dono *et al*, 1996). A RT–PCR methodology was used to detect c-*myc* and glyceraldehyde 3-phosphate dehydrogenase (GAPDH) RNA expression by the leukaemia cells treated in different manners (Cutrona *et al*, 1997).

### Apoptosis assays

Five×10^5^ per ml cells were cultured in RPMI medium (Seromed) supplemented with 10% FCS (Seromed). The cultures were harvested at different intervals and the apoptotic cells were detected by using Annexin-V conjugated with FITC (Apolert^tm^ Apoptosis Kit, Clontech Laboratories Inc., Palo Alto, CA, USA), by PI staining, or by DNA laddering, as previously described (Cutrona *et al*, 1999).

### PNA anti-gene

A 17-mer anti *myc* PNA, complementary to a unique sequence located at the beginning of the second exon of the c-*myc* oncogene (TCA ACG TTA GCT TCA CC) was used. This PNA was modified by addition of a Nuclear Localization Signal peptide (NLS) PNA-myc_wt_-NLS as reported (Cutrona *et al*, 2000). A PNA characterised by a 3-base substitution (T**T**A ACG **C**TA GCT T**T**A CC), but unchanged purine/pirimidine ratio (PNA-myc_mut_-NLS) was used as control. The PNAs described were purchased from TIB MOLBIOL (Berlin, Germany).

### Statistical methods

Mean, standard deviation, and standard error were calculated for each of the markers determined for the two groups of patients investigated. Mean differences by groups of patients were evaluated via the Student's *t*-test and, when necessary (i.e., skewness and/or heteroscedasticity in the biomarker distributions), statistical testing was applied on long transformed variables (Armitage and Berry, 1987). All statistical comparisons and related *P*-values were two tailed. Mean differences were considered significant when *P*-values were less than or equal to 0.05.

## RESULTS

### Definition of CD10-positve and CD10-negative ALL

**Figure 1:**
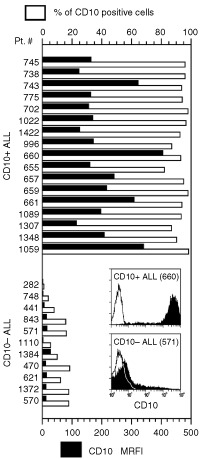
Definition of CD10-positive and CD10-negative ALL. Bone marrow cell suspensions were stained for CD19 and CD10. CD19-positive cells were gated and the percentage of CD10-positive (top scale) cells or the MFRI for CD10 (bottom scale) were recorded. Two typical flow cytometry profiles for CD10 are also shown.

The cells from the bone marrow of 28 ALLs of B cell origin were simultaneously stained for CD10 and CD19. As shown in Figure 1

, the ALL cases were subdivided into two groups: one (17 cases) with a high CD10 expression (>80% CD19-expressing cells were CD10 positive, MRFI 211±82), and one (11 cases) with low CD10 (⩽20% CD19-expressing cells were CD10 positive, MRFI 10±7). Both these differences were highly significant (*P*<0.001 for both percentage positive cells and MRFI values). Cases belonging to the first group, i.e. high CD10 expression, were defined as CD10-positive, while cases falling in the second group, i.e. low CD10 expression, were considered as CD10-negative.

Next, we investigated whether correlations existed in the different cases between the presence of certain chromosome abnormalities and CD10 expression. The 28 ALLs of B cell origin were analysed for the fusion gene transcripts t(1;19) with E2A-PBX1, t(4;11) with MLL-AF4, t(9;22) with BCR–ABL p190 and BCR–ABL p210, t(12;21) with TEL-AML1 by RT–PCR. As shown in Table 1

**Table 1:**
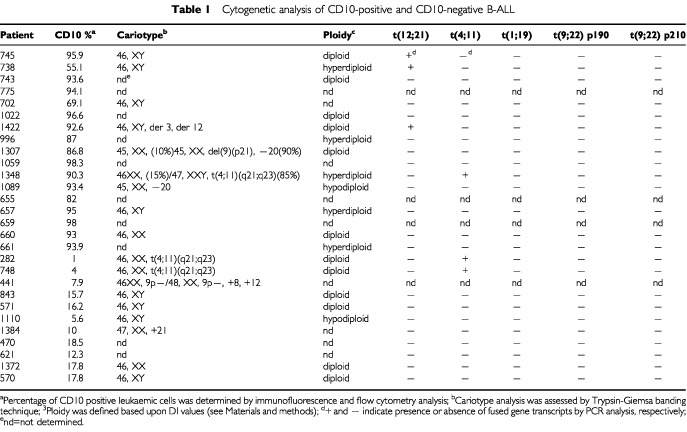
Cytogenetic analysis of CD10-positive and CD10-negative B-ALL

only one out of 14 CD10-positive and two out of 10 CD10-negative ALL cases displayed the t(4;11) translocation, three out of 14 CD10-positive ALL cases displayed t(12;21) rearrangement and t(9;22) was negative in all of the cases analysed. Notably, because of the study design, all of the cases tested did not display any of the typical translocations of BL (Magrath, 1990).

### Differences in spontaneous apoptosis *in vitro*

**Figure 2:**
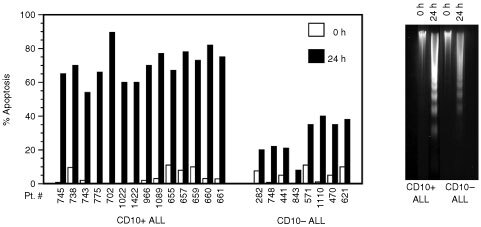
Apoptotic capacities of CD10-positive and CD10-negative ALL cells. Cells from the indicated cases were tested for apoptosis by PI staining either immediately after isolation or following 24 h in culture (left). Apoptotic capacities were confirmed by measuring DNA laddering (two typical experiments are shown on the right).

Here, we investigated whether the malignant cells from CD10-positive ALL had higher spontaneous apoptotic capacities than those from CD10-negative cases. Apoptosis was measured by PI staining in cells taken *ex vivo* or after 24 h in culture. While the proportion of apoptotic cells in the suspensions *ex vivo* was very low in the two groups (mean 4±3.8) (Figure 2

), significant differences were noted after culture. Apoptotic cells in the CD10-positive ALL group were 66.7±9.1% *vs* 22.3±10.5% in the other group (*P*<0.001). These differences were further confirmed by measuring the DNA laddering (Figure 2).

### Differences in cell cycle status

**Figure 3:**
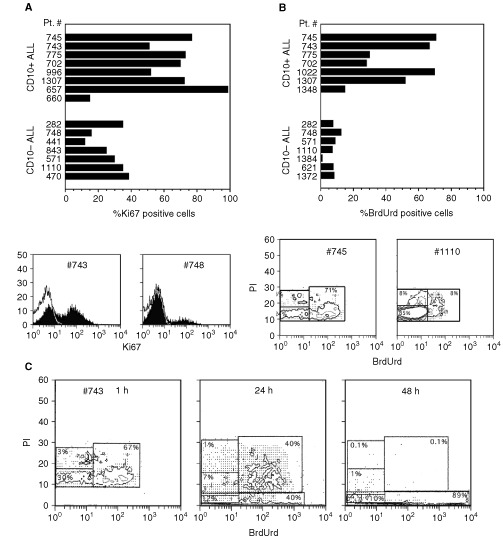
Cell cycle status of CD10-positive and CD10-negative ALL. (**A** and **B**) Cells from the indicated cases were stained for Ki67 (**A**) or BrdUrd (1 h pulse) (**B**) and analysed by flow cytometry. Typical profiles of CD10-positive or CD10-negative ALL cases are shown at the bottom of each figure. (**C**) Cells from a CD10-positive ALL were pulsed with BrdUrd for the indicated times, stained for PI and analysed by flow cytometry. Apoptotic (hypodiploid cells) are observed in the gates at the bottom of the figures.

Next, we investigated the cell cycling abilities of CD10-positive and CD10-negative ALL cells. Eight CD10-positive and seven CD10-negative ALL cases were stained for Ki67, which is expressed from the late G1 phase of the cell cycle (Gerdes *et al*, 1984). The proportion of Ki67-expressing cells was significantly higher in the CD10-positive than in the CD10-negative ALL cases (63.66±23% *vs* 27.37±9.4%, *P*=0.009, see Figure 3A

). Figure 3A also shows typical flow cytometry profiles where a bimodal distribution of Ki67-positive cells is evident for each group.

In another experiment, we investigated BrdUrd incorporation by the CD10-positive and CD10-negative ALL following 1 h in culture. The incorporation of BrdUrd was much higher in the CD10-positive than in the CD10-negative ALLs (average 47.52±21.27% *vs* 7.66±7.84%, *P*=0.001) (Figure 3B). Figure 3B also shows a typical flow cytometry profile obtained in a test where the cells were pulsed with BrdUrd for 1 h, PI stained and analysed by flow cytometry. Most of the cells from the CD10-positive ALL were in the G1-S phase, whereas the majority of the cells from the CD10-negative ALL were in the G0-G1 phase of the cell cycle, a finding which is in agreement with the results of the Ki67 staining.

The cells from five CD10-positive ALL cases (775, 743, 1022, 745 and 657) were pulsed with BrdUrd for different time intervals before PI staining and flow cytometry analysis. Most of the cells were already in the S phase of the cell cycle after 1 h. At later times, there were very few cells in the M phase, whereas the majority of the cells underwent apoptosis from the G1-S phase of the cell cycle, as shown by the finding that virtually all of the hypodiploid cells seen at 48 h had also incorporated BrdUrd (Figure 3C).

### Differences in Myc expression

**Figure 4:**
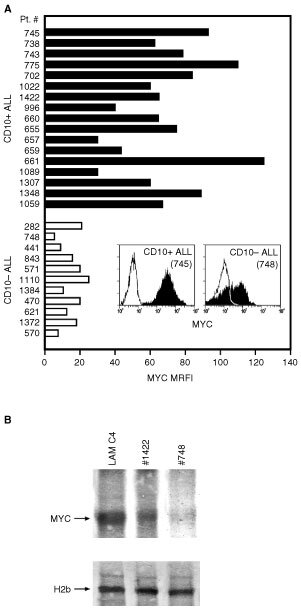
c-*myc* expression by ALL cells. (**A**) Cells from CD10-positive or CD10-negative ALL were stained for MYC protein following permeabilisation (see Materials and methods for details). The fluorescence detected by flow cytometry is expressed as MFRI. Two typical flow cytometry profiles are shown. (**B**) Western blot analysis of Myc expression by the cells from one CD10-positive (#1422) and one CD10-negative (#748) ALL case and by the BL cell line LAM C4. The Western blot was run with purified cell nuclei and probed with an anti Myc or an anti histone 2b antibody. This is a representative experiment of tests carried out on five CD10-positive and four CD10-negative ALL cases.

Cell undergoing apoptosis from the G1-S phase of the cell cycle often overexpress c-*myc* (Hueber and Evan, 1998). To explore this issue further, CD10-positive and CD10-negative ALL were analysed for the expression of Myc protein by immunofluorescence. Single staining was carried out in all cases in which there were greater than 80% neoplastic cells (i.e., CD19^+^/CD10^+^ or CD19^+^/CD10^-^ large blasts), while in two cases, where the proportion of malignant cells was lower (#738 and 702), double staining for surface CD19 and intracytoplasmic Myc was carried out. The data, summarised in Figure 4A

, show a significant difference in Myc expression. CD10 positive ALL cases had an average MRFI of 69.24±25.3 *vs* 14.98±6 of CD10-negative ALL cases (*P*<0.001). These findings were confirmed by Western blot analysis (Figure 4B). The percentages of cells positive for Myc were not determined since the cells of both groups displayed some positive fluorescence, as indicated by the typical profiles shown in the inset of Figure 4A. It could be argued that because of its relatively short half life (Hann and Eisenman, 1984), Myc expression was influenced by the cryopreservation of the cells. However, extensive comparative tests carried out in case 657 demonstrated that cryopreservation did not affect Myc expression. Moreover, cases (655, 657, 659, 660, 661) were also tested using freshly drawn cells.

### Features of CD10-positive cells isolated from CD10-negative ALL cases

**Figure 5:**
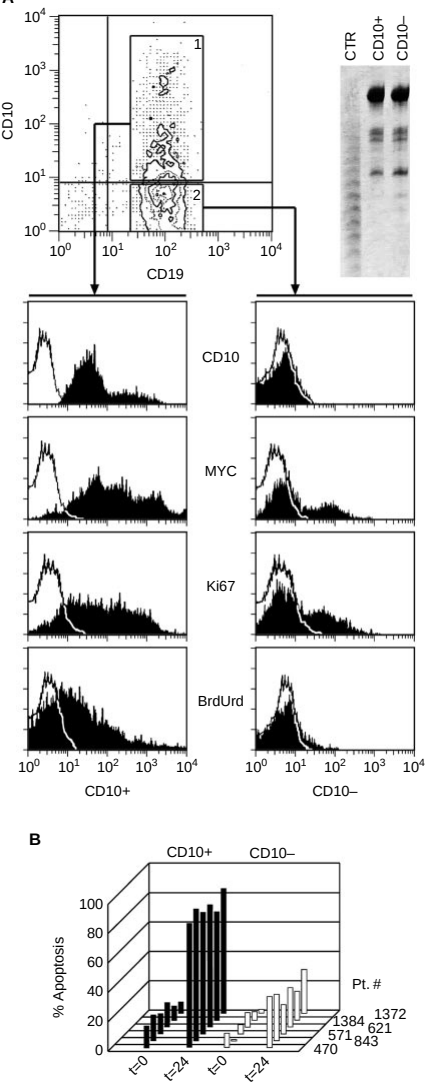
Features of CD10-positive cells isolated from CD10-negative ALL cases. (**A**) Cells from ALL case #571 were stained for CD10 and separated as indicated. Both fractions contained cells from the same clone as assessed by V(D)J length analysis (top right). Cells from the CD10-positive and CD10-negative cell fractions were stained for Myc and Ki67 or incubated with BrdUrd for 24 h before being stained with the appropriate reagents. The stained cells were analysed by flow cytometry. (**B**) The apoptotic capacities of CD10-positive and CD10-negative cell fractions purified from the indicated CD10-negative ALL cases were measured by PI staining before and after 24 h in culture.

Next, we investigated the characteristics of the few CD10-positive cells detected in the CD10-negative ALL cases. The cells from six cases (571, 1372, 621, 1384, 470 and 843) were stained with CD10 mAb and the CD10-positive cells were FACS-sorted. Sufficient quantities of CD10-positive and CD10-negative cells could be isolated for further analyses. As shown in Figure 5

(which reports one representative experiment on cells from patient #571), CD10-positive cells were actively cycling, with high levels of c-*myc* and a special propensity to undergo apoptosis *in vitro*, whereas CD10-negative cells had low c-*myc* levels and low cycling and apoptotic capacities (Figure 5). These data demonstrate a different functional status in two cell populations that originated from the same clone, as shown by the analyses of their V(D)J rearrangements (see Figure 5).

### Role of c-*myc* in apoptosis, cell cycle and CD10 expression

Here, we tested the hypothesis that the high level of c-*myc* conferred to CD10-positive ALL cells both a superior cycling capacity and a more pronounced propensity to undergo apoptosis. To this end, c-*myc* expression was blocked using a PNA anti c-*myc* anti-gene. PNAs are synthetic structural homologues of nucleic acids in which the phosphate-sugar polynucleotide backbone is replaced by a flexible polyamide (Egholm *et al*, 1992). They can bind to the complementary DNA sequences more stably than DNA itself (Egholm *et al*, 1993). However, to be effective on intact cells *in vitro*, PNAs must be coupled to a vector, which allows their transport to the cell nuclei (Nielsen, 1999). In this study, we used PNA with complementarity to a sequence of the second exon of the c-*myc* oncogene. When coupled to an appropriate nuclear localisation sequence (NLS), this PNA-myc_wt_-NLS is able to penetrate cell nuclei and to selectively block the expression of c-*myc* (Cutrona *et al*, 2000). A PNA that differed for the presence of three mutations in the sequence complementary to the c-*myc* sequence was used as control (PNA-myc_mut_-NLS).

**Figure 6:**
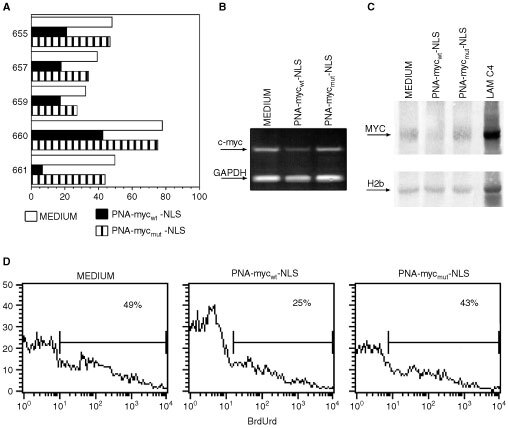
Inhibition of c-*myc* expression by PNA-myc_wt_-NLS. (**A**) The indicated CD10-positive ALL were incubated with PNA-myc_wt_-NLS, PNA-myc_mut_-NLS or medium for 24 h and analysed for Myc expression by flow cytometry. (**B** and **C**) Cells from one representative CD10-positive ALL sample (#661) were cultured for 24 h and analysed for c-myc expression by RT–PCR (**B**) and Western blot (**C**). (**C**) Cells from one CD10-positive ALL case (#657) were treated with the indicated reagents for 24 h, pulsed with BrdUrd for 1 h and analysed by flow cytometry to determine the fraction of the cells in the S phase of the cell cycle.

Cells from CD10-positive ALL were incubated with medium, 10 μM PNA-myc_wt_-NLS, or PNA-myc_mut_-NLS for 24 h. The cultured cells were harvested and checked for c-*myc* expression by flow cytometry (Figure 6A

), RT–PCR (Figure 6B) and Western blot (Figure 6C). As shown in Figure 6, a mean reduction in MRFI of 54±18% was noticed by immunofluorescence and flow cytometry. This reduction in c-*myc* expression was confirmed by the two other methods. Inhibition of c-*myc* expression was accompanied by a significant decrease of BrdUrd incorporation by the cells (Figure 6D), indicating a substantial decline in their cycling abilities.

**Figure 7:**
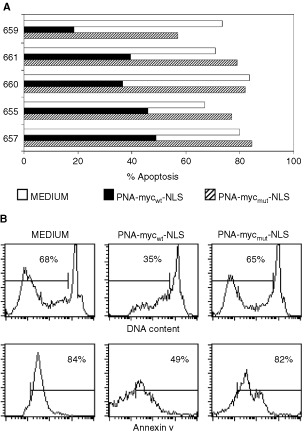
Inhibition of apoptosis in CD10-positive ALL cells by PNA-myc_wt_-NLS. (**A**) Cells from the indicated cases were exposed to the indicated PNAs or to medium alone for 24 h *in vitro*. Apoptosis was then measured by PI or Annexin-V-FITC staining (not shown). (**B**) Apoptotis of cells from case #660 measured by PI or Annexin-V-staining. The cells were exposed to the indicated PNAs for 24 h *in vitro* prior to the test for apoptosis.

In another series of experiments, freshly prepared cells were incubated with PNA-myc_wt_-NLS, PNA-myc_mut_-NLS or medium for 24 h. Subsequently, apoptosis was measured by PI or Annexin-V staining and flow cytometry. A substantial inhibition of apoptosis (mean reduction 49.4±16.8%) was noticed in all of the five cases tested. Figure 7A

shows all of the data obtained by PI staining, which was indicative of both methods used (Figure 7B).

**Figure 8:**
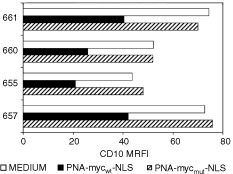
Inhibition of CD10 expression by PNA-myc_mut_-NLS. Cells from the indicated CD10-positive ALL cases were treated with PNAs or medium alone for 24 h in culture and subsequently stained for CD10. Fluorescence was measured by flow-cytometry and expressed as MFRI.

Finally, we checked whether a block in c-*myc* expression caused a concomitant reduction in CD10-expression. As shown in Figure 8

, in the four cases that were investigated, there was a substantial inhibition (mean reduction 48±4.3%) of CD10 expression as assessed by flow cytometry (Figure 8).

## DISCUSSION

This study demonstrates that malignant B cells of CD10-positive ALL are mostly actively cycling, and are apoptosis-prone, whereas the cells of CD10-negative ALL are not actively cycling, and do not undergo apoptosis readily *in vitro*. In addition, the present data show a close correlation between Myc levels and the cycling and apoptotic capacities of the cells. Finally, these studies indicate that CD10 expression distinguishes cells with different properties.

Various results from this study point to correlations between CD10 expression and the cells' biological behaviour *in vitro*. The few CD10-positive malignant cells, present in those cases classified as CD10-negative ALL, shared all of the characteristics of the malignant cells found in the CD10-positive ALL cases, including elevated c-*myc* levels and the propensity to undergo spontaneous apoptosis. Furthermore, the block imposed on c-*myc* expression by PNA myc_wt_-NLS was followed by inhibition of cell cycling, as well as of the apoptotic capacities of the ALL cells. Upon c-*myc* inhibition, there was also a substantial reduction of CD10 expression, thus confirming the value of CD10 as a marker for cycling and/or apoptosing ALL cells.

A wide range of results for Ki67 staining and BrUrd incorporation in ALL cases was reported in previous studies, but these features were not correlated with the immunophenotype of the malignant cells (Tsurusawa *et al*, 1995; Salomons *et al*, 1999).

Admittedly, most of the cases reported could have been CD10-positve. This raises the questions as only in those cases the proliferative capacities of the leukaemic cells were in general lower than those observed for CD10-positive ALL cases in the present study. Although there are no apparent explanations for these differences, one should, however, point out that the flow cytometry method used here may have increased the ability of the techniques to detect cycling cells.

The present study indicates a fundamental role of c-*myc* in the apoptosis of ALL cells, since spontaneous cell death occurred *in vitro* only in those cells that displayed elevated c-*myc* levels. Whether this correlation holds true also for other types of apoptosis (e.g. drug or radiation induced) has to be further investigated, although studies on different cell types overexpressing c-*myc* would predict this to be the case (Rowe *et al*, 1987; Cutrona *et al*, 1995, 1997, 1999; de-Saint-Vis *et al*, 1995; Martinez-Valdez *et al*, 1996; Mueller *et al*, 1997; Guedez *et al*, 1998a,b). A close correlation between c-*myc* expression and apoptosis was first indicated by studies on murine fibroblasts transfected with the c-*myc* oncogene (Evan *et al*, 1992). Recent studies on cells induced to overexpress c-*myc* *in vitro* have demonstrated that these cells have difficulties in completing cell cycle and usually either undergo apoptosis from the late G1 phase or remain arrested in the G2 phase. These arrested cells have a tendency to become aneuploid (often hyperdiploid) (Felsher *et al*, 2000). The latter mechanism observed *in vitro* may suggest an appealing model to explain the frequent hyperdiploidy seen in CD10-positive ALL cells. In this respect, it is of note that hyperdiploidy was detected in 41.7% of the CD10-positive ALL cases studied (five out of 12, Table 1) (see also reviews Look, 1997; Faderl *et al*, 1998; Ito *et al*, 1999).

The finding of elevated c-*myc* levels raises questions regarding the mechanisms involved in c-*myc* overexpression in CD10-positive ALL cells. Chromosomal translocations typical of BL and L3 leukaemias, which juxtapose c-*myc* to the Ig gene loci and cause c-*myc* overexpression, were excluded in all of the ALL cases selected for this study. Therefore, other options have to be considered. One possibility is that, because of the neoplastic transformation, the CD10-positive ALL cells were frozen at a maturational stage characterised by a physiological c-*myc* elevation and active cycling capacities. Studies on murine B cells have demonstrated that under particular circumstances, pre-B cells may proliferate autonomously *in vitro* (Rolink *et al*, 2000) and conceivably also *in vivo* (Carsetti, 2000). Moreover, there is evidence in man that most CD10-positive pre-B cells are actively cycling *in vivo* (Ghia *et al*, 1996). Another plausible, and not mutually exclusive, option is that alterations of oncogenes other than c-*myc* could be responsible for the malignant transformation and for the upregulation of c-*myc* (and cell proliferation). Finally, it is possible that in ALL cells the c-*myc* gene presented a number of mutations which prolong the intracellular half-life of its product, similar to that reported in certain BL cells (Bahram *et al*, 2000; Fais *et al*, 2000). Central to this issue is the problem of whether ALL cells express physiological (i.e. similar to those seen in proliferating cells) or pathological (i.e. similar to levels of those of BL) c-*myc*. Definitive proof for one or other hypothesis awaits further studies.

Previous investigations on cells with elevated c-*myc* levels have indicated that the choice between apoptosis or cell proliferation is often dictated by the presence of additional signals capable of promoting or preventing apoptosis (Bissonnette *et al*, 1992; Fanidi *et al*, 1992). For example, B cells in which the c-*myc* oncogene has been transfected and hyper-expressed would die in low serum concentrations *in vitro* unless exposed to CD40L or to an agonistic CD40 mAb which are both capable of inducing the upregulation of bcl2 and possibly of other anti-apoptotic genes (Cutrona *et al*, 1995). These observations suggest that a number of signals, delivered either by contact with stromal cells or by cytokines, prevent the apoptosis of CD10-positive ALL cells *in vivo* and facilitate their proliferation (Welch *et al*, 1990; Manabe *et al*, 1992, 1994; Saeland *et al*, 1992; Campana *et al*, 1994; Murti *et al*, 1996; Nishigaki *et al*, 1997). In this regard, it is of note that previous studies demonstrated that the ability of ALL cells to survive when cultured on stromal cells was an accurate predictor of a negative clinical outcome irrespective of the cell proliferative ability *in vitro*, possibly underlying the value of the anti-apoptotic properties of the stromal cells in the survival/expansion of the malignant ALL cells (Campana *et al*, 1993; Coustan-Smith *et al*, 1996; Kumagai *et al*, 1996).

Both CD10-positive and CD10-negative ALL are comprised of a heterogeneous group of diseases characterised by different cytogenetic abnormalities. Although data on such abnormalities are not available for all of the cases studied here, observations on a proportion of the CD10-positive ALL points to their heterogeneity. Thus, the t(4;11) translocation, which involves MLL-AF4 gene (Pui *et al*, 1991), was found in only one out of 14 CD10-positive and 2/10 CD10-negative ALL cases. Likewise t(12;21) causing TEL-AML1 (Romana *et al*, 1995; McLean *et al*, 1996; Borkhardt *et al*, 1997) rearrangement was seen in three out of 14 CD10-positive ALL cases.

The latter findings perhaps suggest a marginal involvement of these translocations in the control of c-*myc* expression. On the other hand, the intrinsic properties of the cells from the CD10-negative ALL do not prevent them from expressing CD10 as shown by the data in Figure 5, which reinforced the concept that CD10 expression is linked to the cycling/apoptotic properties. This consideration may in part explain why CD10 expression represents a non-independent prognostic marker of ALL. Thus, the superior cycling and apoptotic features of CD10-positive ALL cells may render them more susceptible to the external signals including chemotherapeutic agents. However, additional features independent of the cycling/apoptotic capacities of the cells may also greatly influence the course of the disease.
